# A Quieter Ocean: Experimentally Derived Differences in Attentive Responses of *Tursiops truncatus* to Anthropogenic Noise Playbacks before and during the COVID-19-Related Anthropause

**DOI:** 10.3390/ani13071269

**Published:** 2023-04-06

**Authors:** Paige E. Stevens, Veda Allen, Jason N. Bruck

**Affiliations:** 1 Department of Integrative Biology, Oklahoma State University, 501 Life Sciences West, Stillwater, OK 74074, USA; 2 Arthur Temple College of Forestry and Agriculture, Stephen F. Austin University, SFA Station, Nacogdoches, TX 75962, USA; 3 Department of Biology, Stephen F. Austin University, SFA Station, Nacogdoches, TX 75962, USA

**Keywords:** anthropause, anthropogenic noise, attention, cognition, noise pollution, cetaceans, dolphins

## Abstract

**Simple Summary:**

The COVID-19 pandemic caused a decrease in human activity in many areas across the globe due to stay-at-home orders and the cessation of non-essential work activities. This dramatic decrease in human activity became known as an anthropause which created an opportunity for scientists to study the effects of decreased anthropogenic (human-made) noise. Bermuda did not host cruise ships or allow non-essential activities from early in the pandemic through to 20 May 2021. We presented three anthropogenic sound types (cruise ship, personal watercraft, and Navy low-frequency active sonar) to dolphins housed at Dolphin Quest Bermuda in 2018 and again during the COVID-19-related anthropause in 2021. We played each dolphin three anthropogenic sound types via an underwater speaker. The dolphins’ behavioral and acoustic responses were recorded. We found that decreased anthropogenic noise levels around Bermuda were associated with altered attention in the dolphins. The dolphins increased look durations and changed vocal response behaviors to playbacks during the anthropause and the dolphins responded to cruise ship playbacks up to fourfold more in 2021. These changes in response to this sound type may have implications on how dolphins habituate and dishabituate to sounds ever-present in their environment.

**Abstract:**

The effects of anthropogenic noise continue to threaten marine fauna, yet the impacts of human-produced sound on the broad aspects of cognition in marine mammals remain relatively understudied. The shutdown of non-essential activities due to the COVID-19-related anthropause created an opportunity to determine if reducing levels of oceanic anthropogenic noise on cetaceans affected processes of sensitization and habituation for common human-made sounds in an experimental setting. Dolphins at Dolphin Quest Bermuda were presented with three noises related to human activities (cruise ship, personal watercraft, and Navy low-frequency active sonar) both in 2018 and again during the anthropause in 2021 via an underwater speaker. We found that decreased anthropogenic noise levels altered dolphin responses to noise playbacks. The dolphins spent significantly more time looking towards the playback source, but less time producing burst pulse and echolocation bouts in 2021. The dolphins looked towards the cruise ship sound source significantly more in 2021 than 2018. These data highlight that different sounds may incur different habituation and sensitization profiles and suggest that pauses in anthropogenic noise production may affect future responses to noise stimuli as dolphins dishabituate to sounds over time.

## 1. Introduction

As humans continue to expand across the globe, anthropogenic noise levels have historically increased, especially in urban areas [1]. Determining the impact of anthropogenic noise has become an essential aspect of conservation as human-produced sounds are increasingly recognized as a global problem [2,3,4,5,6,7] with litigation related to the mitigation of marine anthropogenic noise reaching the United States Supreme Court [8]. These noise levels are not only a potential danger to wild animal populations, but also a potential cause of negative welfare states in managed-care marine species [9]. Major sources of oceanic anthropogenic noise include private and commercial boat traffic, cruise ships, oil industry activities, sonar, and personal watercrafts [1,10]. These oceanic noise sources affect resident and non-resident marine mammal species including cetaceans, such as whales, dolphins, and porpoises. As an indicator species exposed to numerous human-made sounds, common bottlenose dolphins, *Tursiops truncatus* [11], are an ideal species on which to conduct cognition-related anthropogenic noise studies. From these studies, researchers can extrapolate general effects to critically endangered delphinid species that are too imperiled to include in field research and who are not commonly found in managed care facilities. 

### 1.1. Cetaceans and Anthropauses

In 2020, the COVID-19 pandemic interrupted normal operations and changed daily life for the foreseeable future. Multiple countries went into lockdown, ceasing numerous commercial and recreational activities across the globe. This COVID-19-related event was coined as an anthropause [12], but this was not the first time such a thing had happened. The event on 11 September 2001 presented scientists with a short-term anthropause that lasted two days after the tragic events and was confined mostly to North American sea-based shipping traffic. Due to this event, anthropogenic noise in the Bay of Fundy, Canada, dropped by 6 dB with a significant decrease in low-frequency noise under 150 Hz [13]. This change in noise levels was associated with decreased baseline stress levels found in the fecal glucocorticoids of endangered North Atlantic right whales, *Eubalaena glacialis* [14], suggesting anthropogenic noise could be a chronic stressor for the resident animals [13]. While this immediate drop in anthropogenic noise shed some insight into the effects of reduced human activity on marine mammal physiology, the breadth of taxonomic focus in this previous work was limited by time and resources. We have since learned more about cetacean noise responses, which has opened the door for more granular cognitive studies of the relationship noise has with other cetacean groups. Furthermore, as in 2001, 2020 has provided scientists with a natural experimental setup to return to the question of how anthropauses affect cetaceans broadly. 

With a longer duration anthropause and better technology, this most recent period of decreased human activity has created an opportunity for scientists to study the effects of global shutdowns on both terrestrial and marine organisms, and has provided insights on how species respond to sudden abrupt changes in human mobility [12]. During lockdown in areas where scientists were able to complete data collection, researchers found an association between a reduction in human activity and improved air quality, cleaner beaches, and decreased anthropogenic noise [15,16]. Studies completed during the COVID-19 pandemic continue to be published and illuminate the positive and negative effects on species of an extended decrease in human activity across the globe. 

The COVID-19-related anthropause was inconsistent across the globe. Longden et al. [17] found that two areas in Sarasota, FL exhibited different amounts of anthropogenic activity during COVID-19 protocols, with one area receiving 80% higher amounts of vessel activity than pre-pandemic levels. Dolphin whistle detection remained the same at sites with high levels of vessel traffic, indicating that dolphins were not avoiding the area despite increased human presence. However, the detection of dolphin whistles decreased by up to 25% in areas with less vessel traffic [17]. Many urban species in areas with decreased anthropogenic presence experienced positive changes as they moved about differently in their habitat, increased daily activity levels, and rapidly responded to this anthropogenic presence change [18]. Some examples of urban species who increased their movement include wild boars, *Sus scrofa* [14], who descended onto the city of Barcelona, Spain and many Italian towns [19], mountain goats, *Oreamnos americanus* [20], from the Great Orme headland who took over a Welsh seaside resort town when the streets became deserted [18], and Roe deer, *Capreolus capreolus* [14], in Poland who went into cities and towns they had previously avoided [18]. In some urban waterways, anthropogenic noise decreased as much as threefold and created an opportunity for fish and dolphins to increase the distance of their communications by up to 65% [21]. This indicates that the species did not have to increase their call output volumes (the Lombard Effect) [22] and that the decreased masking of noise may have created higher communication efficacy in populations in areas of reduced anthropogenic noise during the anthropause [21].

Bermuda followed suit with many countries regarding COVID-19-related regulations, quarantines, and stay-at-home orders [23]. On 28 March 2020, the first mandatory national lockdown order came into effect [24]. Travel to the island stopped until 1 July 2020. After the island reopened, strict travel and quarantine restrictions remained in place, limiting tourism activities. Most tourist destinations remained closed and cruise ships did not return until the end of May 2021. This dramatic drop in tourism and boat traffic across the globe created quieter oceans, but only in areas with strict quarantine orders [15,16,25,26]. The cessation of these anthropogenic sounds created an opportunity to study the effects of an anthropause on attentive responses in a population that frequently hear anthropogenic noise given they are housed in an open-ocean-type environment. 

### 1.2. Noise and Cetaceans 

Due to a highly specialized auditory system that amplifies sound through mandibular fat channels [27], a brain adapted to process auditory information of the environmental soundscape [28], and the physical optimization of long-range acoustic propagation in an oceanic environment [27,29,30,31,32], dolphins are at risk to be perpetually impacted by anthropogenic sound [33]. Thus, they are potentially at an increased risk of physiological harm, negative welfare states, and cognitive effects as anthropogenic noise increases. Depending on dosage [10,30,34], anthropogenic noise effects vary from sub-lethal to lethal. Physiological changes to cetaceans resulting from anthropogenic noise exposure include hearing loss (temporary or permanent), echolocation changes, stress effects, and death [10,13,30,34,35,36,37]. Hearing loss related to traumatic anthropogenic noise events may even cause consequences as severe as strandings [38]. Communication changes are marked by the Lombard effect as well as modulations of whistle frequency parameters [22,39,40]. Recently, researchers found that dolphins under managed care had to increase their whistle volume in the presence of anthropogenic noise playbacks during cooperation tasks. Furthermore, the success rate of cooperation tasks decreased by up to 20% in tasks performed under the highest noise levels [41]. Behavioral effects in wild settings include avoidance behaviors, and alterations of distributions across space and time [42,43,44]. Studies on physiological changes, distribution changes, and auditory threshold shifts amidst anthropogenic noise pollution are robust, with exposure criteria and recommendations revised frequently [10,34,37,45]. However, studying a multi-year cessation of the modern levels of anthropogenic sound faced by cetaceans has not been possible until the COVID-19 pandemic. 

### 1.3. Habituation and Sensitization

The dual process pathway of habituation and sensitization shapes cognitive responses to excitement or fear-provoking stimuli [46,47]. Sensitization and habituation responses may be deleterious or beneficial depending on behavior and context. For example, dolphins strongly habituated to vessel sound may be at an increased risk of boat strikes [48] but continue to perform survival critical behaviors. Contrarily, dolphins sensitized to anthropogenic sound levels may be at a lower risk of boat strike, but they may decrease the performance of survival critical behaviors, creating a state of elevated metabolic stress [35]. In the presence of noise, the way in which animals habituate or sensitize to sounds can predict the consequence and severity of anthropogenic noise response. Anthropogenic noise sources vary in frequency, duration, and intensity. Due to these variations, habituation and sensitization responses may fluctuate depending on the specific sound stimulus. Habituation responses manifest as a marked decrease in response each time an individual is exposed to a stimulus [47,49]. While many species rapidly habituate to increased human disturbance [50,51,52,53], sensitization may also occur. Sensitization responses are marked by an increase in response to repeated exposures of a stimulus [46,49]. Some migratory bird species have shown increased flight initiation distances with increased human presence [54]. Penguins in New Zealand that previously had a negative exposure to humans (were bled for testing purposes) displayed increased heart rate recovery times when seeing humans compared to their counterparts that did not have such an experience [50]. When measured across a period of two years, resident wild adult dolphins from New Zealand who were exposed to consistent tourism programs exhibited increased avoidance responses to human contact attempts [55]. Yet, inexperienced juveniles did not exhibit the same increased avoidance to these attempts, indicating that the wild adult resident dolphins were sensitizing to the human presence while the wild juveniles had not yet sensitized or habituated [55]. 

Sensitization and habituation are not fixed processes. Changes in levels of noxious or startling stimuli can initiate the disruption of habituation and sensitization effects. Dishabituation is an increase in response to previously habituated stimuli while sensitization involves previously unhabituated stimuli [56]. Additionally, dishabituation involves an “old” stimulus being represented as “new.” Dishabituation to stimuli results in the need for the animal to re-habituate back down to baseline levels to avoid constant disruption and negative impacts on survival critical behaviors. We were able to opportunistically assess whether dishabituation occurs as a function of the extended cessation of organically occurring anthropogenic noise in a real oceanic soundscape using controlled methods afforded by work with animals under human care. Thus, we hypothesized that dolphins re-exposed to anthropogenic sounds that were absent during the pandemic would respond with more attention (investigative behaviors) to experimentally reintroduced presentations of previously familiar anthropogenic noises due to dishabituation effects. Specifically, we hypothesized that dolphins would respond in a similar fashion both before and during the pandemic to the sound of an idling personal watercraft due to the continued presence of this watercraft at their home facility. Similarly, we hypothesized that the dolphins would respond to Navy low-frequency active sonar in the same manner as before the pandemic due to little to no change in sonar exposure in the oceans surrounding Bermuda during the anthropause. Conversely, we hypothesized that dolphins would investigate cruise ship stimuli more due to the sudden absence of cruise ship sound stimuli present near the facility, facilitating a dishabituation effect. 

## 2. Materials and Methods

### 2.1. Setting

**Figure 1:**
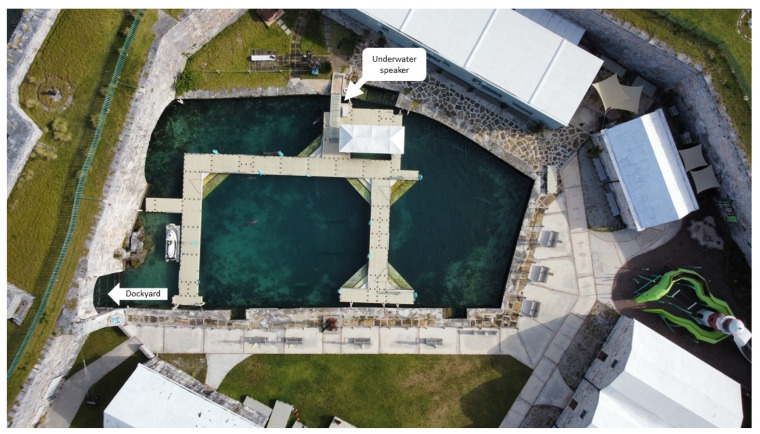
Location of playback trials. The playback speaker location is pictured with the arrow extending to the location below the dock at which the speaker was placed in the water. The white arrow labelled dockyard indicates the ocean gate in which sounds from the port flows through. Photo Credit: Savannah Damiano, Bruck Lab.

The Dolphin Quest Bermuda (DQB) facility was chosen for the close proximity of the animal holding pools to the port in Royal Navy Dockyard, Bermuda. DQB consists of pools with open water access to the ocean by a gate allowing sounds from the port to enter the pools. Cruise ships dock beyond the rock walls at the Kings Wharf in direct line with the opening to the ocean (see Figure 1). Pools consist of natural substrate and depths fluctuate with the tide. 

### 2.2. Subjects

To assess the amount of disruption in dolphin attention, 13 bottlenose dolphins (*n* = 13; 11 females and 2 males, F ages 1–45, M aged 1 and 12) were exposed to anthropogenic sounds at DQB in 2018 and 2021 (*n* = 10 in 2018, *n* = 12 in 2021). There were three dolphins born between 2018 and 2021 and the transfer of one dolphin out of the facility during the same time period limiting data collection on them to only 2018. Two dolphins were born at the beginning of the COVID-19 pandemic and one dolphin was born the summer before the pandemic. This limited their sound exposure to a few months before a strict quarantine disrupted normal anthropogenic sound levels. The new dolphins received the same number of tests as all other dolphins in 2021 (*n* = 9 per dolphin). All other dolphins were tested individually 18 times (*n* = 18 per dolphin). 

### 2.3. Noise Exposure

As a result of anthropogenic activities in the ocean waters that feed into their lagoon, the dolphins at DQB hear common anthropogenic sounds, such as docking and departing cruise ships, commercial boat traffic moving through the sound, and personal watercrafts. Cruise ships port and leave multiple times per week with a day in between docking. In 2018, five different contracted cruise ships docked for a combined total of 106 docking events across the year while 41 other non-contracted cruise ships docked for a combined total of 74 events with as many as three cruise ships at port at once [57]. During the busy 2020 pre-COVID season, cruise ships docked between 23–26 times [57] and the subjects were exposed to personal watercraft eight times per day as an island touring company operated on the wharf in Royal Navy Dockyard. Additionally, the dolphins were exposed to DQB personal watercraft operations, however, speed was limited to idling when the personal watercraft was returning to the facility each time. Cruise ships stopped docking at the island in March of 2020 and did not return until the end of May 2021 [58]. Their absence served as an indicator of the length of the anthropause. During the anthropause, the dolphins were exposed to DQB’s personal watercraft on the days it needed to be moved to a mechanic, no cruise ships, minimal marine traffic, and few visitors.

The ambient noise levels of the lagoons as well as anthropogenic noise activities were recorded. The ambient noise levels of the dolphin habitat during the anthropause were on average 47.76 dB and 51.815 dB under normal anthropogenic conditions. Anthropogenic noise stimuli were recorded at distances in which they would hear the sounds. One of the contracted cruise ships, Celebrity Summit, was recorded at the Royal Navy Dockyards in Bermuda with a maximum sound level volume of 76.21 dB at the dolphin’s habitat. We presented the cruise ship sound at an average maximum of 73.66 dB intensity which closely matched the amplitude of sound experienced by the dolphins in their habitat. The idling personal watercraft was recorded at a maximum of 85.35 dB in the dolphin’s lagoon, and we presented the sounds at 83.76 dB from 1 m away (see Section 2.4 for recording procedures). To our knowledge, no Navy activity occurred near Dolphin Quest that would necessitate the use of Low-Frequency Active Sonar (LFAS) of the type we used as a playback stimulus. Past Navy sonar playback studies have played at sound intensities between 115 dB and 185 dB, however, these studies used a mid-frequency active sonar and one study was measuring the endocrine responses of bottlenose dolphins [59]. In order to not cause a response due to a sound intensity shift or initiate a stress response, we played the Navy LFAS sound at levels that were congruent with the other playbacks at an average sound intensity of 75.29 dB. All source level intensities were calculated with Praat 6.1.48 from a hydrophone placed at the underwater speaker sound source (see Section 2.4 for recording equipment). 

### 2.4. Playback Presentation

**Figure 2:**
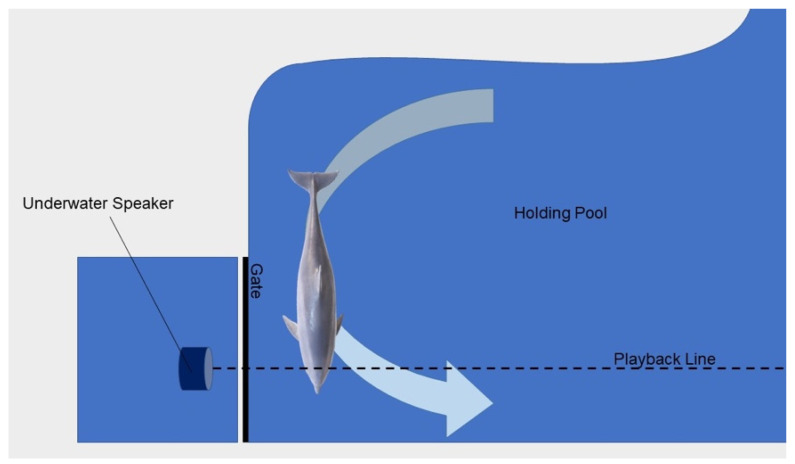
Underwater playback schematic. In the playback paradigm, the sound source is played from an underwater Lubell Labs™ LL16 speaker 0.5–1.0 m from the dolphin’s head to ensure appropriate playback sound levels to the subjects. The sounds are initiated when the target dolphin self-separates from conspecifics and crosses the playback line. Swim direction is indicated with the arrow.

To assess the effects of reintroduced anthropogenic noise after a long-term anthropause, dolphins at DQB, Royal Naval Dockyard, Bermuda were presented with control and anthropogenic sounds via an established underwater playback methodology [60,61] (see Figure 2). Procedures consisted of setting up an underwater speaker and a two-hydrophone array. Playbacks were initiated when the target dolphin self-separated from conspecifics while moving, and crossed an invisible playback triggering line (Figure 2). This procedure makes the behavioral responses overt and easy to score. During the playbacks, the dolphins were recorded with a camera until 20 s after they ceased to respond to the initial sound stimuli source by look.

We performed playbacks three times per day, every allowable weather day, across two weeks per year of the study. Playbacks were calibrated to be audible without causing harm and match source levels of anthropogenic noise from outside the dolphin enclosures [60,61]. The team presented trial sets composed of two control sounds designed to habituate the response levels to a zero-response baseline and then an experimental sound (anthropogenic noise stimuli) from 0.5 m to 1 m. Control sounds included a cut piece of a whistle fragment (control A) and a 10 kHz pure test tone (control B). The control sounds were played in a specific order with the 10 kHz control B test tone played initially. This is a sound that is the same frequency as a trainer bridge/whistle. Control B was played first then we played the control A whistle fragment sound (see [56,57] for calibration procedures). These controls have been used with this group of dolphins for 15 years, and they allow us to gauge the animal’s interest before the experimental playback. Additionally, we are able determine if baseline dolphin response levels to sounds are generally the same in each trial from these controls [60,61]. If the target dolphin did respond with look to these control playbacks, then an attempt was made to habituate the individual to speaker sound by repeated presentations of the eliciting control sound spaced apart by 5 min. This was ultimately successful in all cases, however, the response given to the first presentation of a control sound was used in the analysis with control A serving as the intercept for our models (see Section 2.7). 

**Figure 3:**
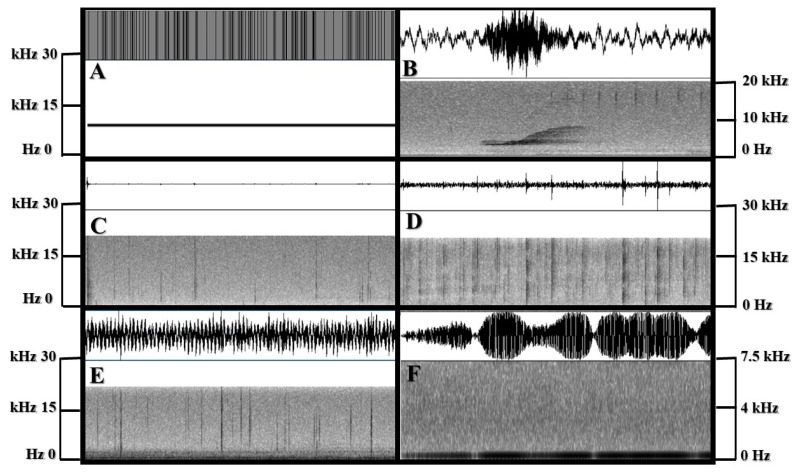
Spectrograms of Playback Sounds (Hz). Except for the 10 kHz Control B tone (1 s), all windows are 3 s in duration. (**A**) Control B: 10 kHz test tone. (**B**) Control A: dolphin whistle fragment. (**C**) Ambient soundscape of dolphin pools without added anthropogenic sound. (**D**) Cruise ship. (**E**) Idling personal watercraft. (**F**) Navy LFAS.

After control presentations, each dolphin was presented with one of three anthropogenic noise types per playback session (for a total of three sessions per dolphin): a mid-sized cruise ship leaving port (Celebrity Summit: one of the five contracted cruise ships that returns to the island frequently; 100 Hz–1 kHz), Navy low-frequency active sonar (Navy LFAS; 100 Hz–0.5 kHz), and a Sea-Doo GTI (hereafter referred to as idling personal watercraft; 100 Hz–10 kHz) (see Figure 3). Noises were presented at the same output level to match normal environmental levels for these subjects as they experienced them (see Section 2.3). The sequence of the anthropogenic noise presentations was randomized per dolphin to limit the possibility of order effects. Each anthropogenic noise except the Navy LFAS was recorded via a SENSOR Technology Ltd. (Collingwood, ON, Canada) Sea Phone SQ26-08 omnidirectional hydrophone (sensitivity −168.8 dB re 1 V/µPa; flat frequency response from 100 Hz to 30 kHz, ±3 dB), with a bandwidth between 20 Hz and 50 kHz recorded to a ZOOM H1N-VP recorder (Hauppauge, NY, USA). The cruise ship and idling personal watercraft sounds were recorded at the wharf in Royal Navy Dockyard, Bermuda, until the vessel was absent and then cut down into 3 s increments. The cruise ship was recorded from approximately 100 m from the source and 3 m in depth and the idling personal watercraft was recorded at a distance of 1 m away and 1 m in depth. The final sound, Navy LFAS, was obtained via a sound sharing website online (https://dosits.org/galleries/audio-gallery/anthropogenic-sounds/surtass-lfa-sonar-sound/ (accessed on 20 April 2018)). One exemplar per sound type was used as the playback stimuli. 

As the target dolphin self-separated from conspecifics and crossed the threshold or playback line, playbacks were initiated by a seventh generation Apple Inc. iPod Touch™ (Los Altos, CA, USA) (Figure 2). Each trial was video recorded above and below water with underwater audio directly channeled to a Canon™ (Ohta-ku, Tokyo, Japan; FS200) camera until 20 s after the target dolphin stopped responding to the playback. Underwater footage was recorded on a GoPro 6 (San Mateo, CA, USA). We recorded vocalizations during the trials via two SENSOR Technology Ltd. (Collingwood, ON, Canada) Sea Phone SQ26-08 omnidirectional hydrophones (sensitivity −168.8 dB re 1 V/µPa; flat frequency response from 100 Hz to 30 kHz, ±3 dB), with a bandwidth between 20 Hz and 50 kHz recorded to a PreSonus (Baton Rouge, LA, USA) 2-channel AudioVox^®^ USB (frequency: 48 kHz) connected to a 2018 Apple Inc.^®^ Macbook Pro (Los Altos, CA, USA) or a 2020 HP Inc.^®^ Envy x360 convertible (Palo Alto, CA, USA) running Audacity version 2.3.3 [62] software. The playbacks were projected from a Lubell Labs™ (Columbus, OH, USA; LL916) underwater speaker (0.6–21 kHz ± 8 dB) connected to a Hertz^®^ amplifier (Electromedia; Potenza Picena, Italy; HCP 2) powered by a 12 V battery. The audio was calibrated to be audible without initiating a startle response [63]. 

The effects of the anthropause resulting from the COVID-19 pandemic on dolphin attention were assessed by returning to the DQB facility in May 2021 and presenting the same anthropogenic noises played in 2018 via the same playback methodology (see Figure 2) and equipment as in 2018.

### 2.5. Behavior Scoring

**Figure 4:**
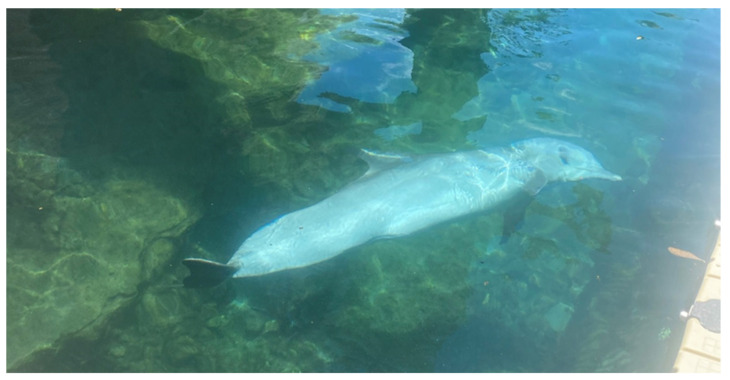
Dolphin Response. A look response to a playback. The underwater speaker source is behind a gate directly in line with the dolphin’s gaze.

After the data collection was completed, trials were scored for frequency and duration of looks (see Figure 4). A total of 214 trials were completed (*n* = 214), however, sessions with video missing or interrupted by storms were not included for scoring (*n* = 204). Looks were chosen because dolphins do not always approach a sound source and may investigate without approaching closely. Three scorers independently and blindly tallied behaviors for each recording in Boris behavioral software 7.12.2 [64]. Scorers were trained by the same person before evaluating any videos on their own and each scored the entire data set. Behavioral scores that differed by greater than 20% required all three of the observers to come to a consensus on one score. If the scorers did not differ more than 20%, the three observer scores were averaged for those data points. It was planned that any score not agreed upon was sent to a fourth mediator for the final decision; however, this plan was never utilized as all scorers were able to come to a consensus.

### 2.6. Acoustic Analysis

**Figure 5:**
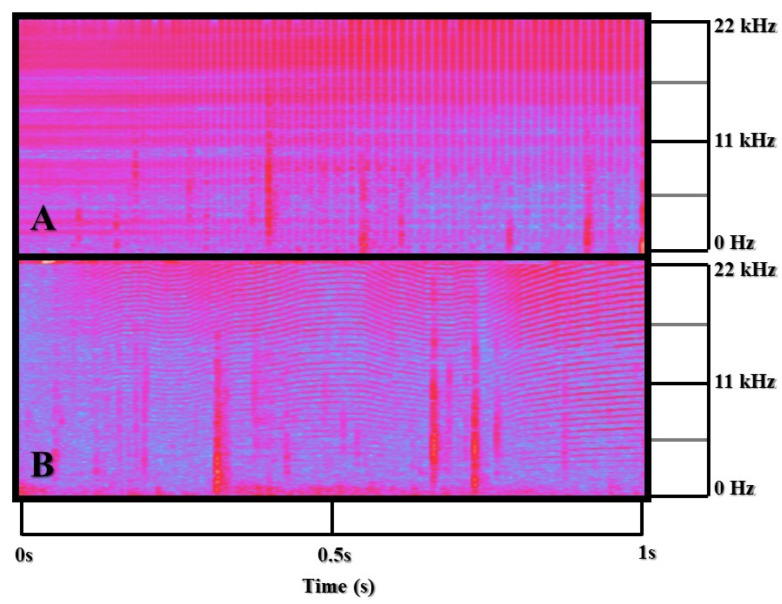
Echolocation bout and burst pulse spectrograms (Hz). (**A**) Spectrogram of an echolocation click train. (**B**) Spectrogram of a burst pulse response with clear harmonic features. The vertical bands shown in red are snapping shrimp present in the natural soundscape.

A comprehensive audio analysis was performed for each trial by two independent blind observers using Audacity 3.0.0 [62]. Given the volume of data, observers worked on separate files after a training period and an interobserver reliability check. The two observers tallied the duration of echolocation and burst pulse vocalizations across 203 trials (*n* = 203) which equated to about 10.15 h of time. Echolocation and burst pulse vocalizations both consist of broadband clicks that extend ultrasonically but differ by their interpulse intervals [65] (see Figure 5 for echolocation and burst pulse vocalization spectrograms). Burst pulses were defined by interpulse clicks less than 10 ms apart with a clearly defined start and stop [65,66] that were not a part of any echolocation click train. Additionally, burst pulses are often delineated by harmonic features. Observers had to have a 90% or better interobserver reliability for ten 10 min training files before they completed any individual analysis on the acoustics. This reliability was calculated by dividing the identified durations of burst pulse and echolocation bouts by the total time of the session (which could not deviate by more than 10%). This led to two aggregate reliability measures for the duration of bouts for both echolocation and burst pulse sounds. Echolocation was chosen to gauge interest in and exploration of the sound stimuli. Burst pulse vocals are a communicative signal between dolphins [67,68] and were initially used as a potential measure of aggression.

### 2.7. Statistical Analysis

To test differences in behavioral and acoustic responses by year and playback type, we built Generalized Linear Mixed Models (GLMMs) in RStudio 2021.09.01 [69] using the lme4 package to run [70] and the fitdistrplus package for distribution fitness [71]. For each model, we used the test subject (target dolphin) as a random effect variable to allow for interindividual variation across behavioral responses. Fixed effects for each model included year and playback stimuli. Each model was first tested for fitness of data distribution via the fitdistrplus R package and plotted with Q-Q plots and Cullen and Frey graphs to visualize fitness. Subsequently, we used GLMM models with a gamma family and log function to test whether there was a significant difference in look, echolocation, and burst pulse duration responses by year and playback type. Control A was used as the intercept control for the GLMM tests so that all stimuli were compared to baseline levels. Additionally, we checked for an effect of new dolphins on results by running a separate analysis without the young and relocated dolphins that mirrored our results of zero dolphins taken out of the study.

## 3. Results

### 3.1. Behavioral Results

**Figure 6:**
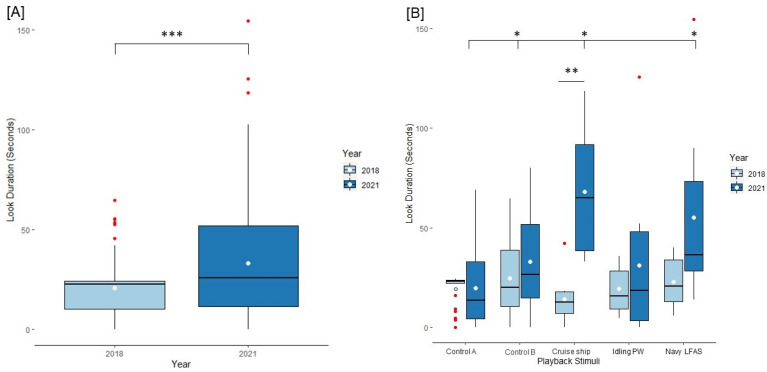
Look duration responses broken down by playback stimuli and year. (**A**) Boxplots representing the interquartile range of look responses (seconds) by year. Outlier points are included in red. The means of each group are represented by the white circles in each box. (**B**) Boxplots illustrating the interquartile range of look duration responses across playback files and year. The top line with an asterisk denotes significance across playbacks and the lower line without endcaps denotes significance for a pairwise comparison across years. The dot that indicates the mean for *control A* is outlined with a black line. * = *p* < 0.05, ** = *p* < 0.01, *** = *p* < 0.001. Idling PW = idling personal watercraft.

Overall, the dolphins showed significantly more interest through look responses to anthropogenic noise playbacks during the anthropause in 2021 than in 2018 (Generalized Linear Mixed Model (look duration: (2018) $\bar{x}$ = 20.92 ± 1.54 s, (2021) $\bar{x}$ = 33.17 ± 2.75 s, t = 18.232, *p* < 0.001; see Figure 6a)). Additionally, dolphins responded differently by playback type. Relative to control A, look duration responses varied significantly for control B, cruise ship, and Navy LFAS (GLMM (intercept $\bar{x}$ = 19.51 ± 2.78 s, z = 18.021 ± 0.154 s, *p* < 0.001; control B $\bar{x}$ = 28.84 ± 3.67 s, z = 2.109 ± 0.161, *p* = 0.0349; cruise ship $\bar{x}$ = 41.22 ± 6.63 s, z =2.488 ± 0.285, *p* = 0.0128; idling personal watercraft $\bar{x}$ = 25.23 ± 7.55 s, *p* = 0.36; Navy LFAS $\bar{x}$ = 45.54 ± 8.86 s, z = 2.386 ± 0.294, *p* = 0.0107; see Figure 6b)). There was a significant interaction effect between playback and the year of playback of the cruise ship sound on look duration (t = 2.797 ± 0.552, *p* = 0.005).

### 3.2. Acoustic Results

**Figure 7:**
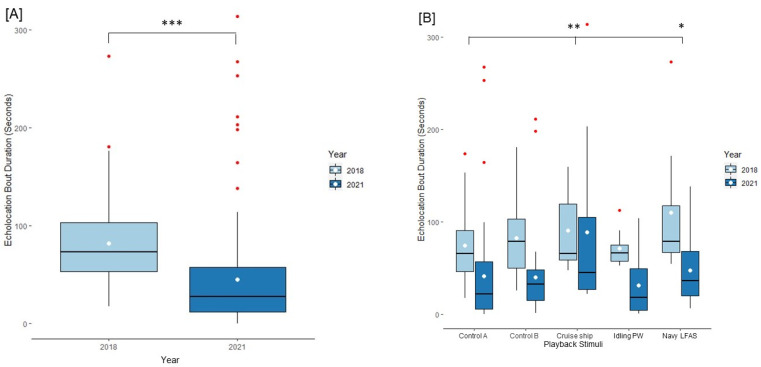
Echolocation Bout Duration Responses separated by fixed effect factors. (**A**) Box and whisker plot representing the IQR and medians of echolocation duration responses in 2018 and 2021 visits. Means are represented by white dots. (**B**) Box and whisker plot representing data range of echolocation durations by playback type with outliers denoted in red. * = *p* < 0.05, ** = *p <* 0.01, *** = *p* < 0.001. Idling PW = idling personal watercraft.

**Figure 8:**
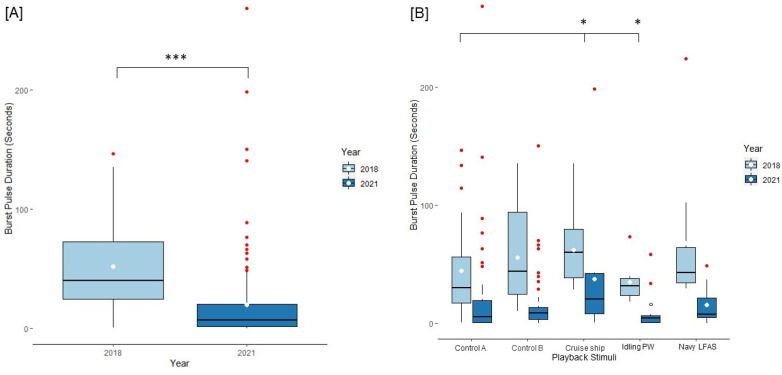
Burst Pulse bout Duration Reponses separated by fixed effect factors. (**A**) Box and whisker plot of decreases in burst pulse duration responses across the years 2018 and 2021. (**B**) Burst pulse duration responses grouped by year and playback type. * = *p* < 0.05, *** = *p* < 0.001. Means are denoted with white circles and the Idling personal watercraft is given with a black outline around the white circle. Outliers are denoted by red dots. Idling PW = idling personal watercraft.

We tested for acoustic responses by evaluating duration of echolocation bouts and burst pulse durations. Echolocation and burst pulse duration responses were significantly different between the years 2018 and 2021 (echolocation duration: 2018 $\bar{x}$ = 82.22 ± 4.79 s; 2021 $\bar{x}$ = 44.86 ± 4.95 ss seconds, t = −7.576, *p* < 0.001; burst pulse duration: 2018 $\bar{x}$ = 52.00 ± 4.2 s, 2021 $\bar{x}$ = 19.927 ± 3.41 s, t = −8.670, *p* < 0.001; see Figure 7a and Figure 8a). Playback type was a significant factor for two echolocation duration responses including cruise ship and Navy LFAS (2018: intercept $\bar{x}$ = 74.20 ± 7.49 s, z = 25.006, *p* < 0.001; control B $\bar{x}$ = 82.32 ± 7.49 s, z = 1.289, *p* = 0.197; cruise ship $\bar{x}$ = 90.5 ± 12.57 s, z = 2.988, *p* = 0.002; idling personal watercraft $\bar{x}$ = 71.35 ± 7.27 s, z= −0.947, *p* = 0.34; Navy LFAS $\bar{x}$ = 109.99 ± 23.64 s, z= 2.175, *p* = 0.030; 2021: intercept $\bar{x}$ = 44.86 ± 4.95 s, control B $\bar{x}$ = 39.86 ± 6.13 s, cruise ship $\bar{x}$ = 73.43 ± 27.87 s, idling personal watercraft $\bar{x}$ = 31.55 ± 10.67 s, Navy LFAS $\bar{x}$ = 47.27 ± 11.49 s; Figure 7b). Furthermore, burst pulse duration responses were significantly different for the cruise ship, and idling personal watercraft sounds relative to control A (2018: intercept $\bar{x}$ =19.93 ± 6.98 s, z = 17.356, *p* < 0.001; control B $\bar{x}$ = 22.68 ± 6.48 s, z = 0.588, *p* = 0.55; cruise ship $\bar{x}$ = 23 ± 10.47 s, z = 2.316, *p* = 0.021; idling personal watercraft $\bar{x}$ = 17.5 ± 6.15 s, z= −2.537, *p* = 0.011; Navy LFAS $\bar{x}$ = 68.09 ± 20.97 s, z = 0.951, *p* = 0.341; 2021: intercept $\bar{x}$ = 44.69 ± 4.01 s, control B $\bar{x}$ = 16.64 ± 4.09 s, cruise ship $\bar{x}$ = 43.75 ± 16.89 s, idling personal watercraft $\bar{x}$ = 10.53 ± 5.623 s, Navy LFAS $\bar{x}$ = 15.46 ± 4.83 s; Figure 8b). There was not a significant interaction effect between playback type and year for either echolocation duration or burst pulse duration.

### 3.3. Results of Dolphins Tested across Both Years

When tested with only dolphins tested across both years included, the results remained significant across the same playback types for look duration response (GLMM (intercept t = 13.312, *p* < 0.001; Year t = 2.134, *p* = 0.033; control B t = 3.208, *p* < 0.01, cruise ship t = 2.020, *p* = 0.04, idling personal watercraft t = 0.434, *p* = 0.67, Navy LFAS t = 3.451, *p* < 0.001)). Additonally, we found that echolocation duration were significantly different across multiple playback files (GLMM (intercept z = 29.25, *p* < 0.001; Year z = −8.345, *p* < 0.001; control B z = 2.264, *p* = 0.024; cruise ship z = 3.028, *p* = 0.002; idling personal watercraft z = −0.668, *p* = 0.50, Navy LFAS z = 3.047, *p* = 0.002)). Finally, we found that burst pulse duration responses were significantly different between the years tested (GLMM (intercept z = 16.607, *p* < 0.001; Year z = −6.002, *p* < 0.001, control B z = 0.991, *p* = 0.32; cruise ship z = 0.949, *p* = 0.343; idling personal watercraft z = −0.727, *p* = 0.467, Navy LFAS z = 1.086, *p* = 0.277)).

## 4. Discussion

### 4.1. COVID-19 Anthropause and Investigative Behaviors

Our study sought to measure the effects of the months-long cessation of anthropogenic noise due to the COVID-19 pandemic on common bottlenose dolphin cognition, specifically attention and habituation. We predicted that dolphins would present longer investigative behaviors during the anthropause in 2021 than in 2018 due to broad dishabituation effects. Specifically, we predicted that cruise ship responses would exhibit the greatest change because of the lockdown orders and the cessation of cruise ship porting. Overall, the dolphins looked on average significantly longer during the anthropause than before (Figure 6a). This indicates that the sudden onset of anthropogenic noise after a long-term cessation may create a period where the dolphins must again habituate to the presence of elevated levels of human noise. Habituation characteristically becomes a shorter process with each recurrence of a non-startling stimuli [72]; thus, any successive habituation training may take less time for the dolphins to get back to pre-pandemic levels. However, the potential negative effects of dishabituating, such as increased responses to the eliciting stimuli and decreased time spent executing survival critical behaviors may present after an anthropause as animals readjust to human activity. These results could be combined with stress and sound avoidance data taken from wild populations where we are not able to perform habituation and dishabituation studies, to paint a broader picture of how cognition, stress, and noise interact to affect the welfare of wild populations. Additionally, not all areas around the world exhibited decreased anthropogenic noise [17], thus, there will be a variation of consequences of re-introduced noise on populations across the globe.

As predicted, we found a significant association between look behaviors and the year of data collection for cruise ship playbacks. Dolphins responded to the cruise ship stimulus by looking at the sound source up to fourfold longer during the anthropause than in 2018 (Figure 6b). The same was not true for personal watercraft (which had decreased but had repeated presentations during the anthropause) or Navy LFAS (which likely saw no change in its already decreased rate both before and during the anthropause). The studied dolphins did not exhibit sensitization to any of the playbacks, but there was a dishabituation pattern to cruise ships. Abrupt cessation of normal anthropogenic activity interrupted the almost-daily conditioning during peak season of cruise ships by dolphins at DQB. It should be noted that longer response durations do not imply increased stress responses. While unstudied, the possibility of dishabituation possibly extends to other populations, such as resident bottlenose dolphins in Galveston Bay off the coast of Texas that live in a high-traffic shipping channel [73]. However, to our knowledge, these Galveston Bay resident dolphins were unable to be studied during the anthropause. This type of dishabituation could negatively impact dolphins as a lost habituation response may lead to greater distractibility and stress at the reapplication of the potentially disruptive sound source. While the anthropause related to COVID-19 was unprecedented and unexpected, these results allow us to extrapolate the effects of abrupt noise cessation and its reintroduction on dolphins in areas with strict quarantine orders. However, dolphins in areas with less severe quarantine restrictions may not exhibit a dishabituation response. As humans move into previously abandoned areas, more studies on post-anthropause anthropogenic noise responses are necessary. With the introduction of uncertain and non-uniform responses to COVID-19 and other potential factors resulting in anthropauses, it is critical to factor in wildlife responses to abrupt changes in human activities at all scales.

Additionally, we found that behavioral responses varied significantly by playback type. Dolphins spent significantly more time investigating the low-frequency noise types, namely the *cruise ship* and the relatively uncommon (for them) Navy LFAS, than the higher frequency sounds (Figure 6b). While this may be a function of the relative infrequency of Navy LFAS in these animals’ lives along with the dishabituation effect of cruise ships, this is one of the few studies to identify an increased response to low-frequency sounds in a small cetacean relative to high-frequency sounds [9]. Low-frequency noise is one of the most prominent and problematic anthropogenic noises due to the pervasive nature of low-frequency sound propagation and the types of vessels and activities that produce low-frequency noise [1]. While the dolphins tested in our study sought out the anthropogenic noise source, not all dolphins respond similarly. The increased presence of cruise ships over a decade have shifted Hector’s dolphin (*Cephalorhynchus hectori* [74]) distributions towards the dolphin’s normal outer habitat away from the inner harbor where cruise ships dock [75]. This may be due to the presence of cruise ships themselves more so than the noise levels. Another common low-frequency anthropogenic noise, *Navy LFAS*, in particular has been linked to consequences as severe as hearing loss [32,76] and stranding deaths [38,77]. Higher frequency noises were either not as distracting as the Navy LFAS and cruise ship sounds or not significant as was the case for an idling personal watercraft. However, personal watercrafts moving at full speed have a greater modulation and a higher upper-frequency range that may impact dolphin attentive responses differently. Additionally, the DQB dolphins are not harassed by the proximity of personal watercrafts in the same way as many wild dolphins in popular recreation areas. Thus, it would not be fully appropriate to draw conclusions about the response profile of waterjet-propelled personal watercraft without testing the sound of one accelerating at full throttle.

### 4.2. Anthropause and Acoustic Response

Under the normal anthropogenic noise conditions of 2018, cruise ships and personal watercrafts periodically and frequently raised the ambient sound levels by ~30 dB, whereas the pool soundscape was not raised by anthropogenic noises during the anthropause. Our acoustic results showed that dolphins produced shorter bouts of echolocation and burst pulse durations in 2021 than 2018 (Figure 7a and Figure 8a). Another communication study with dolphins conducted during the COVID-19 pandemic found anthropogenic activity levels in areas of Sarasota Bay, FL remained consistent or even increased as much as 80% with dolphin whistle detection remaining consistent in areas of increased vessel activities [17]. Yet, whistle detection rates decreased in areas of less vessel traffic [17]. From Buckstaff [78], we know that vessel traffic leads to increased dolphin vocalizations on vessel approach. The decreased detection of dolphins’ whistles in areas of less vessel presence may be due to reduced whistle presence rather than decreased dolphin presence. With regard to masking, in New Zealand waterways, dolphin communication ranges increased during the anthropause by 1 km [21]. This means that in low noise environments we should expect clearer sound transmission channels and, if all things are equal, the detection of more vocalizations. We found that echolocation bouts were significantly shorter in duration during the anthropause than before. Therefore, our subjects were vocalizing less in low noise environments, as would be expected from the aforementioned studies. The decreased echolocation durations of DQB dolphins during 2021 may be due to decreased masking effects resulting in more efficient acoustic exploration rather than decreased interest. They did not investigate via echolocation as long, but they displayed increased behavioral interest as measured by longer look durations.

Burst pulse click trains have been linked to aggressive interactions between bottlenose dolphins [68,79], hence we expected to see an increase in burst pulse production during the 2021 playbacks as we expected to see an increased rate of aggression as a function of more salient anthropogenic noise playbacks. Across multiple species, increased aggression resulted from additional anthropogenic noise. Anemone fish (*Amphiprion chrysopterus* [80]) exposed to intermittent motorboat noise stayed closer to their anemone and responded with increased territorial aggression toward heterospecifics [81]. When anthropogenic noise increased in rural territories, the European Robin (*Erithacus rubecula* [14]) displayed an increase in aggression [82]. Urban robins responded with more behaviors that are aggressive to simulated intrusions. However, urban robins did not display an increase in aggression when anthropogenic noise levels increased as their rural counterparts did. When tested during the COVID-19 anthropause, our subjects decreased the duration of their burst pulses (Figure 8a), which may be a parallel to the processes seen in urban robins. More likely, however, is that this reduction in burst pulse signals may be a better measure of conspecific attention away from each other and towards the playbacks. Future studies could focus on the effects of anthropogenic noise levels on aggression between dolphin conspecifics in environments across high and low levels of anthropogenic noise. Additionally, research on displaced and conspecific aggression during an anthropause could illuminate potential social effects for facilities that are open ocean or wild dolphins living in areas with large variations in anthropogenic noise levels across seasons. However, it is likely that dolphin aggression may not be shaped by noise as readily as we expected.

### 4.3. Limitations

We are confident that we have experimentally identified a dishabituation effect to anthropogenic noise occurring in a natural soundscape, however, there are some caveats to our study. One area of interest not addressed by this study is the duration of dishabituation vulnerability. We cannot say how long a wild dolphin might be vulnerable to a resumed noxious sound source after their previous habituation. Nor can we determine how anthropauses of various lengths affect dishabituation responses. Future studies may address the former by evaluating mortality or cortisol changes directly after an anthropause where noise varies. Another limitation of this opportunistic study is that the stimuli chosen in 2018 were for another hypothesis and therefore do not represent a large cross-section of the potential sounds a dolphins may experience in the wild [9]. Therefore, while we highlight low-frequency noise responses as interesting in this study, we do not have the cross-section of different types of playback sounds (nor the sample size for various replicants of these sounds) to make broader assessments on how sound characteristics relate to overall responses or how sound type might affect dishabituation profiles. Furthermore, it should be noted that the facility transferred one dolphin from 2018 and had three births in between retesting pre-COVID. Two more calves were tested in 2021 than 2018 due to these changes, but the mean age of the population remained about the same in both years. With subjects as a random effect, we are confident that our model has captured the phenomena in question. However, we ran an additional statistical test with the only the dolphins that received playbacks across both years with subjects as a random variable as well. This test presented similar significance across all variables; thus, we retained the dolphins in the data presented.

## 5. Conclusions

Studies over the long-term anthropause related to COVID-19 are continuing to be published, but the effects of human absence and the sudden reintroduction of human noise are still understudied. This is due to the difficulty of accessing study subjects during times of society-wide limits on human activity. The strict lockdown orders by the Bermudian government provided an opportunity to gather baseline data on anthropogenic noise responses after a months-long cessation of widely encountered noises, such as the sounds of cruise ships. This revealed that abrupt changes in ambient anthropogenic noise levels may affect previously habituated responses by dolphins. These data are some of the first to address the effects of various anthropogenic noise types on dolphin attention, how dolphin responses change during the cessation of anthropogenic activity, and how particular low-frequency anthropogenic noise sources may instigate stronger responses from dolphins in some scenarios. Our results highlight that we must consider how we re-enter the world after a period of isolation both at the scale of the COVID-19 global disruption, but also at local levels where habitats that humans have abandoned become re-populated again.

## Data Availability

The data presented in this study are available upon request from the corresponding authors.

## References

[1] Duarte C.M., Chapuis L., Collin S.P., Costa D.P., Devassy R.P., Eguiluz V.M., Erbe C., Gordon T.A.C., Halpern B.S., Harding H.R. (2021). The soundscape of the Anthropocene ocean. Science.

[2] Perry C. A review of the impact of anthropogenic noise on cetaceans. Proceedings of the Scientific Committee at the 50th Meeting of the International Whaling Commission.

[3] Williams R., Wright A.J., Ashe E., Blight L.K., Bruintjes R., Canessa R., Clark C.W., Cullis-Suzuki S., Dakin D.T., Erbe C. (2015). Impacts of anthropogenic noise on marine life: Publication patterns, new discoveries, and future directions in research and management. Ocean Coast. Manag..

[4] McGregor P.K., Horn A.G., Leonard M.L., Thomsen F. (2013). Anthropogenic noise and conservation. Animal Communication and Noise.

[5] Weilgart L.S. (2007). The impacts of anthropogenic ocean noise on cetaceans and implications for management. Can. J. Zool..

[6] Tyack P.L., Thomas L. (2019). Using dose–response functions to improve calculations of the impact of anthropogenic noise. Aquat. Conserv. Mar. Freshw. Ecosyst..

[7] Erbe C., Dunlop R., Dolman S. (2018). Effects of noise on marine mammals. Effects of Anthropogenic Noise on Animals.

[8] Zirbel K., Balint P., Parsons E. (2011). Navy sonar, cetaceans and the US Supreme Court: A review of cetacean mitigation and litigation in the US. Mar. Pollut. Bull..

[9] Stevens P.E., Hill H.M., Bruck J.N. (2021). Cetacean Acoustic Welfare in Wild and Managed-Care Settings: Gaps and Opportunities. Animals.

[10] Southall B.L., Nowacek D.P., Bowles A.E., Senigaglia V., Bejder L., Tyack P.L. (2021). Marine Mammal Noise Exposure Criteria: Assessing the Severity of Marine Mammal Behavioral Responses to Human Noise. Aquat. Mamm..

[11] Montagu G. (1821). Description of a species of Delphinus, which appears to be new. Mem. Wernerian Nat. Hist. Soc..

[12] Rutz C., Loretto M.-C., Bates A.E., Davidson S.C., Duarte C.M., Jetz W., Johnson M., Kato A., Kays R., Mueller T. (2020). COVID-19 lockdown allows researchers to quantify the effects of human activity on wildlife. Nat. Ecol. Evol..

[13] Rolland R.M., Parks S.E., Hunt K.E., Castellote M., Corkeron P.J., Nowacek D.P., Wasser S.K., Kraus S.D. (2012). Evidence that ship noise increases stress in right whales. Proc. R. Soc. B Biol. Sci..

[14] Linnaeus C.v. (1758). Systema Naturae.

[15] Zambrano-Monserrate M.A., Ruano M.A., Sanchez-Alcalde L. (2020). Indirect effects of COVID-19 on the environment. Sci. Total Environ..

[16] Ihsan Y.N., Purba N.P., Faizal I., Anya A., Mulyani P.G., Anwar S.K. (2022). Impact of the Pandemic COVID-19 to the Indonesia Seas. Geo J. Tour. Geosites.

[17] Longden E.G., Gillespie D., Mann D., McHugh K.A., Rycyk A.M., Wells R., Tyack P.L. (2022). Comparison of the marine soundscape before and during the COVID-19 pandemic in dolphin habitat in Sarasota Bay, FL. J. Acoust. Soc. Am..

[18] Miraglia N., Di Brita A. (2022). Behavior of Wildlife Species in Urban Areas to Changing Conditions during COVID-19 Lockdowns: A Review. J. Appl. Anim. Welf. Sci..

[19] Sharma P., Kaur M., Narwal G. (2020). Other side of the COVID-19 Pandemic: A review. Pharma Innov..

[20] de Blainville H.M.D. (1816). Prodrome d’une nouvelle distribution sytématique du règne animal. Bull. Des Sci. Par La Société Philomatique De Paris.

[21] Pine M.K., Wilson L., Jeffs A.G., McWhinnie L., Juanes F., Scuderi A., Radford C.A. (2021). A Gulf in lockdown: How an enforced ban on recreational vessels increased dolphin and fish communication ranges. Glob. Chang. Biol..

[22] Kragh I.M., McHugh K., Wells R.S., Sayigh L.S., Janik V.M., Tyack P.L., Jensen F.H. (2019). Signal-specific amplitude adjustment to noise in common bottlenose dolphins (*Tursiops truncatus*). J. Exp. Biol..

[23] Murphy M.M., Jeyaseelan S.M., Howitt C., Greaves N., Harewood H., Quimby K.R., Sobers N., Landis R.C., Rocke K., Hambleton I.R. (2020). COVID-19 containment in the Caribbean: The experience of small island developing states. Res. Glob..

[24] Shahid M.A.H., Sarker M.S.A., Hasan M.M., Nasrin T. (2020). COVID-19 Pandemic Lockdown: Be a Reference for Global Environment. IJRASET.

[25] Tyack P., Miksis-Olds J., Ausubel J., Urban E. (2021). Measuring ambient ocean sound during the COVID-19 pandemic. EOS.

[26] Thomson D.J., Barclay D.R. (2020). Real-time observations of the impact of COVID-19 on underwater noise. J. Acoust. Soc. Am..

[27] Au W.W.L., Hastings M.C. (2008). Auditory Systems of Marine Animals. Principles of Marine Bioacoustics.

[28] Popov V.V., Supin A.Y. (2007). Analysis of auditory information in the brains of cetaceans. Neurosci. Behav. Physiol..

[29] Ketten D. Functional analyses of whale ears: Adaptations for underwater hearing. Proceedings of the OCEANS’94.

[30] Ketten D.R., Kastelein R.A., Thomas J.A., Nachtigall P.E. (1995). Estimates of blast injury and acoustic trauma zones for marine mammals from underwater explosions. Sensory Systems of Aquatic Mammals.

[31] Ketten D.R. (1997). Structure and function in whale ears. Bioacoustics.

[32] Mooney T.A., Nachtigall P.E., Castellote M., Taylor K.A., Pacini A.F., Esteban J.-A. (2008). Hearing pathways and directional sensitivity of the beluga whale, Delphinapterus leucas. J. Exp. Mar. Biol. Ecol..

[33] Nachtigall P.E., Supin A.Y., Pacini A.F., Kastelein R.A. (2018). Four odontocete species change hearing levels when warned of impending loud sound. Integr. Zool..

[34] Southall B.L., Finneran J.J., Reichmuth C., Nachtigall P.E., Ketten D.R., Bowles A.E., Ellison W.T., Nowacek D.P., Tyack P.L. (2019). Marine mammal noise exposure criteria: Updated scientific recommendations for residual hearing effects. Aquat. Mamm..

[35] Dey M., Krishnaswamy J., Morisaka T., Kelkar N. (2019). Interacting effects of vessel noise and shallow river depth elevate metabolic stress in Ganges river dolphins. Sci. Rep..

[36] Farmer N.A., Baker K., Zeddies D.G., Denes S.L., Noren D.P., Garrison L.P., Machernis A., Fougères E.M., Zykov M. (2018). Population consequences of disturbance by offshore oil and gas activity for endangered sperm whales (*Physeter macrocephalus*). Biol. Conserv..

[37] Southall B.L., Bowles A.E., Ellison W.T., Finneran J.J., Gentry R.L., Greene C.R., Kastak D., Ketten D.R., Miller J.H., Nachtigall P.E. (2007). Structure of the noise exposure criteria. Aquat. Mamm..

[38] Mann D., Hill-Cook M., Manire C., Greenhow D., Montie E., Powell J., Wells R., Bauer G., Cunningham-Smith P., Lingenfelser R. (2010). Hearing loss in stranded odontocete dolphins and whales. PLoS ONE.

[39] De Clerck S., Samarra F.I., Svavarsson J., Mouy X., Wensveen P. (2019). Noise influences the acoustic behavior of killer whales, *Orcinus orca*, in Iceland. Proc. Meet. Acoust..

[40] Luís A.R., Couchinho M.N., dos Santos M.E. (2014). Changes in the acoustic behavior of resident bottlenose dolphins near operating vessels. Mar. Mammal Sci..

[41] King S.L., Connor R.C., Krützen M., Allen S.J. (2021). Cooperation-based concept formation in male bottlenose dolphins. Nat. Commun..

[42] Castellote M., Brotons J.M., Chicote C., Gazo M., Cerdà M. (2015). Long-term acoustic monitoring of bottlenose dolphins, *Tursiops truncatus*, in marine protected areas in the Spanish Mediterranean Sea. Ocean Coast. Manag..

[43] Kassamali-Fox A., Christiansen F., May-Collado L.J., Ramos E.A., Kaplin B.A. (2020). Tour boats affect the activity patterns of bottlenose dolphins (*Tursiops truncatus*) in Bocas del Toro, Panama. PeerJ.

[44] Papale E., Alonge G., Grammauta R., Ceraulo M., Giacoma C., Mazzola S., Buscaino G. (2020). Year-round acoustic patterns of dolphins and interaction with anthropogenic activities in the Sicily Strait, central Mediterranean Sea. Ocean Coast. Manag..

[45] Southall B., Johnson C., Scholik A., Adam T., Harrison J., Hollinshead K. (2008). US regulation of the effects of sound on marine life: NOAA’S mandates and use of scientific information. Bioacoustics.

[46] Stevens P.E., Bruck J.N., Vonk J., Shackelford T. (2019). Sensitization. Encyclopedia of Animal Cognition and Behavior.

[47] Groves P.M., Thompson R.F. (1970). Habituation: A dual-process theory. Psychol. Rev..

[48] Martinez E., Stockin K. (2013). Blunt trauma observed in a common dolphin delphinus sp. Likely caused by a vessel collision in the Hauraki Gulf, New Zealand. Pac. Conserv. Biol..

[49] Thompson R.F., Berry S.D., Rinaldi P.C., Berger T.W. (2021). Habituation and the orienting reflex: The dual-process theory revisted. The Orienting Reflex in Humans.

[50] Ellenberg U., Mattern T., Seddon P.J. (2009). Habituation potential of yellow-eyed penguins depends on sex, character and previous experience with humans. Anim. Behav..

[51] Vincze E., Papp S., Preiszner B., Seress G., Bókony V., Liker A. (2016). Habituation to human disturbance is faster in urban than rural house sparrows. Behav. Ecol..

[52] Uchida K., Blumstein D.T. (2021). Habituation or sensitization? Long-term responses of yellow-bellied marmots to human disturbance. Behav. Ecol..

[53] Stankowich T. (2008). Ungulate flight responses to human disturbance: A review and meta-analysis. Biol. Conserv..

[54] Blumstein D.T. (2014). Attention, habituation, and antipredator behaviour: Implications for urban birds. Avian Urban Ecol..

[55] Constantine R. (2001). Increased avoidance of swimmers by wild bottlenose dolphins (*Tursiops truncatus*) due to long-term exposure to swim-with-dolphin tourism. Mar. Mammal Sci..

[56] Marcus E.A., Nolen T.G., Rankin C.H., Carew T.J. (1988). Behavioral dissociation of dishabituation, sensitization, and inhibition in Aplysia. Science.

[57] Department of Marine and Ports Services. Government of Bermuda. 2018 Cruise Ship Schedule. 17 May 2018. http://www.rccbermuda.bm/Documents/Shipping_schedules/2018_Cruise_Ship_Schedule.pdf.

[58] Department of Marine and Ports Services. Government of Bermuda. 2021 Cruise Ship Schedule. 16 April 2021. http://rccbermuda.bm/Documents/Shipping_schedules/2021%20Cruise%20Ship%20Schedule.pdf.

[59] Houser D.S., Martin S., Crocker D.E., Finneran J.J. (2020). Endocrine response to simulated US Navy mid-frequency sonar exposures in the bottlenose dolphin (*Tursiops truncatus*). J. Acoust. Soc. Am..

[60] Bruck J.N. (2013). Decades-long social memory in bottlenose dolphins. Proc. R. Soc. B Biol. Sci..

[61] Bruck J.N., Walmsley S.F., Janik V.M. (2022). Cross-modal perception of identity by sound and taste in bottlenose dolphins. Sci. Adv..

[62] Audacity T. (2017). Audacity. The Name Audacity (R) Is a Registered Trademark of Dominic Mazzoni. http://audacity.sourceforge.net.

[63] Fish J.F., Turl C.W. (1976). Acoustic Source Levels of Four Species of Small Whales.

[64] Friard O., Gamba M. (2016). BORIS: A free, versatile open-source event-logging software for video/audio coding and live observations. Methods Ecol. Evol..

[65] Lammers M.O., Au W.W. (2003). Directionality in the whistles of Hawaiian spinner dolphins (*Stenella longirostris*): A signal feature to cue direction of movement?. Mar. Mammal Sci..

[66] Jones B., Zapetis M., Samuelson M.M., Ridgway S. (2020). Sounds produced by bottlenose dolphins (*Tursiops*): A review of the defining characteristics and acoustic criteria of the dolphin vocal repertoire. Bioacoustics.

[67] Blomqvist C., Amundin M. (2004). High-frequency burst-pulse sounds in agonistic/aggressive interactions in bottlenose dolphins, *Tursiops truncatus*. Echolocation in Bats and Dolphins.

[68] Overstrom N.A. (1983). Association between burst-pulse sounds and aggressive behavior in captive Atlantic bottlenosed dolphins (*Tursiops truncatus*). Zoo Biol..

[69] Chambers J.M. (2008). Software for Data Analysis: Programming with R.

[70] Bates D., Mächler M., Bolker B., Walker S. (2014). Fitting linear mixed-effects models using lme4. arXiv.

[71] Delignette-Muller M.L., Dutang C. (2015). fitdistrplus: An R package for fitting distributions. J. Stat. Softw..

[72] Rankin C.H., Abrams T., Barry R.J., Bhatnagar S., Clayton D.F., Colombo J., Coppola G., Geyer M.A., Glanzman D.L., Marsland S. (2009). Habituation revisited: An updated and revised description of the behavioral characteristics of habituation. Neurobiol. Learn. Mem..

[73] Ronje E., Whitehead H., Barry K., Piwetz S., Struve J., Lecours V., Garrison L., Wells R.S., Mullin K.D. (2020). Abundance and occurrence of common bottlenose dolphins (*Tursiops truncatus*) in three estuaries of the Northwestern Gulf of Mexico. Gulf Caribb. Res..

[74] Van Beneden P.J. (1881). Notice sur un nouveau dauphin de la Nouvelle-Zélande. Bull. Roy. Acad. Belg..

[75] Carome W., Slooten E., Rayment W., Webster T., Wickman L., Brough T., Dawson S.M. (2022). A long-term shift in the summer distribution of Hector’s dolphins is correlated with an increase in cruise ship tourism. Aquat. Conserv. Mar. Freshw. Ecosyst..

[76] Mooney T.A., Nachtigall P.E., Vlachos S. (2009). Sonar-induced temporary hearing loss in dolphins. Biol. Lett..

[77] Filadelfo R., Mintz J., Michlovich E., D’Amico A., Tyack P.L., Ketten D.R. (2009). Correlating military sonar use with beaked whale mass strandings: What do the historical data show?. Aquat. Mamm..

[78] Buckstaff K.C. (2004). Effects of watercraft noise on the acoustic behavior of bottlenose dolphins, *Tursiops truncatus*, in Sarasota Bay, Florida. Mar. Mammal Sci..

[79] Caldwell M.C., Caldwell D.K. (1967). Intraspecific transfer of information via the pulsed sound in captive odontocete cetaceans. Animal Sonar Systems: Biology and Bionics.

[80] Cuvier G., Valenciennes A. (1844). Histoire naturelle des poisons. Tome dix-septième. Suite du livre dix-huitième. Cyprinoïdes.

[81] Mills S.C., Beldade R., Henry L., Laverty D., Nedelec S.L., Simpson S.D., Radford A.N. (2020). Hormonal and behavioural effects of motorboat noise on wild coral reef fish. Environ. Pollut..

[82] Önsal Ç., Yelimlieş A., Akçay Ç. (2022). Aggression and multi-modal signaling in noise in a common urban songbird. Behav. Ecol. Sociobiol..

